# Common variant of *BCAS3* is associated with gout risk in Japanese population: the first replication study after gout GWAS in Han Chinese

**DOI:** 10.1186/s12881-018-0583-z

**Published:** 2018-06-07

**Authors:** Masayuki Sakiyama, Hirotaka Matsuo, Hirofumi Nakaoka, Yusuke Kawamura, Makoto Kawaguchi, Toshihide Higashino, Akiyoshi Nakayama, Airi Akashi, Jun Ueyama, Takaaki Kondo, Kenji Wakai, Yutaka Sakurai, Ken Yamamoto, Hiroshi Ooyama, Nariyoshi Shinomiya

**Affiliations:** 1 Department of Integrative Physiology and Bio-Nano Medicine, National Defense Medical College, 3-2 Namiki, Tokorozawa, Saitama, 359-8513 Japan; 2 Department of Dermatology, National Defense Medical College, Tokorozawa, Japan; 3 Division of Human Genetics, Department of Integrated Genetics, National Institute of Genetics, Mishima, Japan; 4 Program in Radiological and Medical Laboratory Sciences, Pathophysiological Laboratory Sciences, Nagoya University Graduate School of Medicine, Nagoya, Japan; 5 Department of Preventive Medicine, Nagoya University Graduate School of Medicine, Nagoya, Japan; 6 Department of Preventive Medicine and Public Health, National Defense Medical College, Tokorozawa, Japan; 7 Department of Medical Chemistry, Kurume University School of Medicine, Kurume, Japan; 8 Ryougoku East Gate Clinic, Tokyo, Japan

**Keywords:** Breast carcinoma amplified sequence 3 (BCAS3), Potassium voltage-gated Channel subfamily Q member 1 (KCNQ1), Regulatory factor X3 (RFX3), Single nucleotide polymorphisms (SNP), Urate, Uric acid

## Abstract

**Background:**

Gout is a common disease resulting from hyperuricemia which causes acute arthritis. A recent genome-wide association study (GWAS) of gout identified three new loci for gout in Han Chinese: regulatory factor X3 (*RFX3*), potassium voltage-gated channel subfamily Q member 1 (*KCNQ1*), and breast carcinoma amplified sequence 3 (*BCAS3*). The lack of any replication studies of these three loci using other population groups prompted us to perform a replication study with Japanese clinically defined gout cases and controls.

**Methods:**

We genotyped the variants of *RFX3* (rs12236871), *KCNQ1* (rs179785) and *BCAS3* (rs11653176) in 723 Japanese clinically defined gout cases and 913 controls by TaqMan method. rs179785 of *KCNQ1* is also evaluated by direct sequencing because of difficulties of its genotyping by TaqMan method.

**Results:**

Although the variants of *RFX3* and *BCAS3* were clearly genotyped by TaqMan method, rs179785 of *KCNQ1* was not, because rs179785 (A/G) of *KCNQ1* is located at the last nucleotide (“A”) of the 12-bp deletion variant (rs200562977) of *KCNQ1*. Therefore, rs179785 and rs200562977 of *KCNQ1* were genotyped by direct sequencing in all samples. Moreover, by direct sequencing with the same primers, we were able to evaluate the genotypes of rs179784 of *KCNQ1* which shows strong linkage disequilibrium with rs179785 (*D’* = 1.0 and *r*^*2*^ = 0.99). rs11653176, a common variant of *BCAS3*, showed a significant association with gout (*P* = 1.66 × 10^− 3^; odds ratio [OR] = 0.80); the direction of effect was the same as that seen in the previous Han Chinese GWAS. Two variants of *KCNQ1* (rs179785 and rs179784) had a nominally significant association (*P* = 0.043 and 0.044; OR = 0.85 and 0.86, respectively), but did not pass the significance threshold for multiple hypothesis testing using the Bonferroni correction. On the other hand, rs200562977 of *KCNQ1* and rs12236871 of *RFX3* did not show any significant association with gout.

**Conclusion:**

BCAS3 is a coactivator of estrogen receptor alpha, and the influence of estrogen to serum uric acid level is well known. Our present replication study, as did the previous gout GWAS, demonstrated the common variant of *BCAS3* to be associated with gout susceptibility.

## Background

Gout, which can also cause acute arthritis, is a common disease resulting from hyperuricemia. An increasing number of patients nowadays suffer from gout. Although various investigations aiming to elucidate the pathogenesis of this common disease are being conducted worldwide, most of the common genetic causes of gout remain to be clarified. Previous function-based genetic studies [1–3] and genome-wide association studies (GWASs) [4–6] have revealed that gout is associated with several genes, such as ATP-binding cassette transporter, subfamily G, member 2 (*ABCG2/BCRP*) and glucose transporter 9 (*GLUT9/SLC2A9*). Especially, by performing a GWAS of clinically-ascertained gout, our Japanese report identified five gout loci including *MYL2-CUX2* and cornichon family AMPA receptor auxiliary protein 2 (*CNIH-2*) [6]. Subsequent fine mapping analysis of the *MYL2-CUX2* region found that rs671 of aldehyde dehydrogenase 2 (*ALDH2*) is a gout locus which is an Asian specific one [7]. Li et al. recently performed a GWAS of clinically- ascertained gout and identified the following three new loci for gout in Han Chinese: regulatory factor X3 (*RFX3*), potassium voltage-gated channel subfamily Q member 1 (*KCNQ1*) and breast carcinoma amplified sequence 3 (*BCAS3*) [8]. However, there is no replication study of these three loci using other population groups. We therefore performed a replication study using Japanese clinically-defined gout cases and controls.

## Methods

### Patients and controls

This study was approved by the institutions’ Ethical Committees (National Defense Medical College and Nagoya University). All procedures were performed in accordance with the Declaration of Helsinki, with written informed consent obtained from each subject. The cases comprised 723 gout patients assigned from Japanese male outpatients at Ryougoku East Gate Clinic (Tokyo, Japan). All patients were clinically diagnosed with primary gout according to the criteria established by the American College of Rheumatology [9]. Patients with inherited metabolic disorders, including Lesch–Nyhan syndrome and phosphoribosylpyrophosphate synthetase I superactivity, were excluded. Hyperuricemia was defined as the serum uric acid (SUA) level that exceeds 7.0 mg/dl (= 416.36 mol/l) according to the guideline of the Japanese Society of Gout and Nucleic Acid Metabolism [10]. The control group comprised 913 Japanese males without hyperuricemia and gout history, recruited from the participants in the Daiko Study, part of the Japan Multi-Institutional Collaborative Cohort Study (J-MICC Study) [11]. The mean age (± SD) of case and control groups was 45.5 years (± 10.6) and 53.5 years (± 10.3), respectively, and their mean body mass index was 25.3 kg/m^2^ (± 3.6) and 22.9 kg/m^2^ (± 2.9), respectively.

### Genotyping

Genomic DNA was extracted from whole peripheral blood cells [12]. Genotyping of the three single nucleotide polymorphisms (SNPs), rs12236871 of *RFX3*, rs179785 of *KCNQ1* and rs11653176 of *BCAS3*, was performed using the TaqMan method (Thermo Fisher Scientific, Waltham, MA, USA) employing a LightCycler 480 (Roche Diagnostics, Mannheim, Germany) [12] with minor modifications. For genotyping *KCNQ1*variants, DNA sequencing analysis was performed with following primers: forward 5’-ACTTCCTGCCTCTGCTTTC-3′ and reverse 5’-TGAAGGAAGTGACCCCTG-3′. Direct sequencing was performed using a 3130xl Genetic Analyzer (Thermo Fisher Scientific) [12].

### Data analysis

The software R version 3.1.1 (http://www.r-project.org/) [13] with the GenABEL package was used for all calculations in the statistical analysis. The association analyses were examined using the chi-square test. The pairwise linkage disequilibrium (LD) was calculated using data from the 1000 Genomes phase 3 JPT (Japanese in Tokyo) [14]. All *P* values were two-tailed and *P* values of < 0.05 were regarded as statistically significant.

## Results

**Fig. 1:**
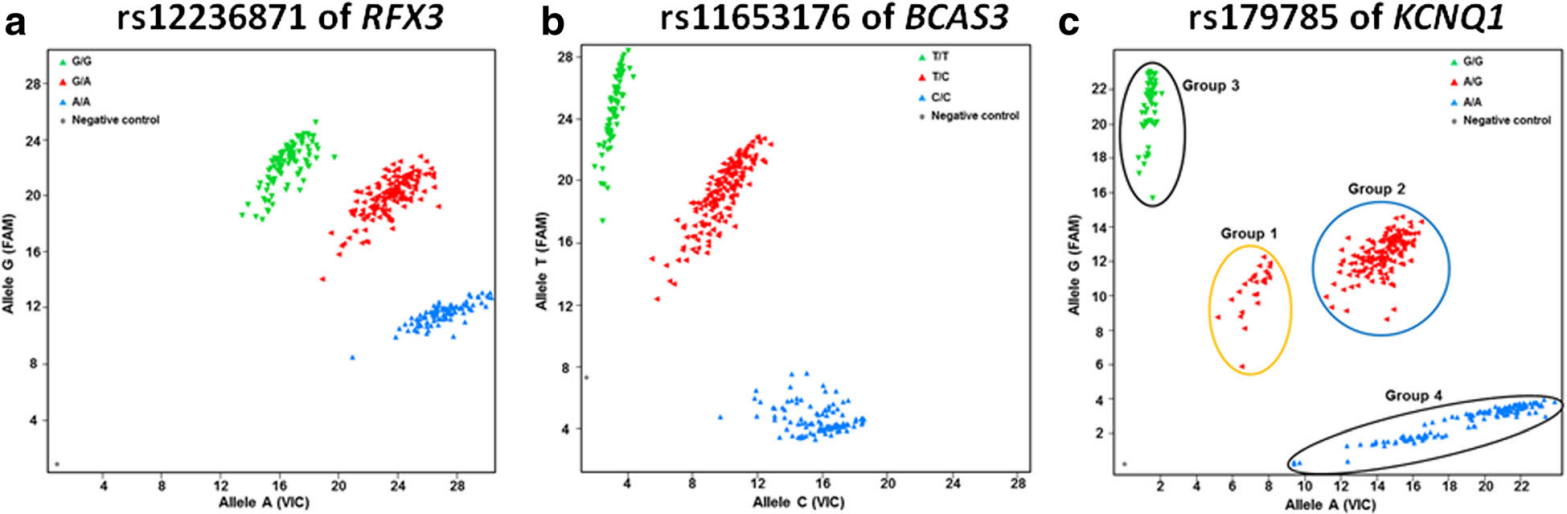
Allelic discrimination plots of SNPs of *RFX2*, *BCAS3* and *KCNQ1*. **a** Representative plots for rs12236871 of *RFX3*. Well-separated clusters representing each genotype (G/G, G/A and A/A) can be observed. **b** Representative plots of rs11653176 of *BCAS3*. Well-separated clusters are clearly visible, representing each genotype (T/T, T/C and C/C). **c** Representative plots of rs179785 of *KCNQ1*. Computer auto analysis divided these plots into three groups (G/G, A/G and A/A). However, the plots seemed to be clustered into four groups (labeled as Groups 1 to 4). We therefore employed direct sequencing to confirm the genotypes. As a result, a 12-bp deletion variant of *KCNQ1* (rs200562977) was identified in several samples of all of four groups, and rs179785 of *KCNQ1* is located at the last nucleotide of this deletion variant (also see Figs. 2 and 3). Therefore, because it is difficult to genotype rs179785 using the TaqMan method, we performed the subsequent genotyping of rs179785 and rs200562977 by direct sequencing

**Fig. 2:**
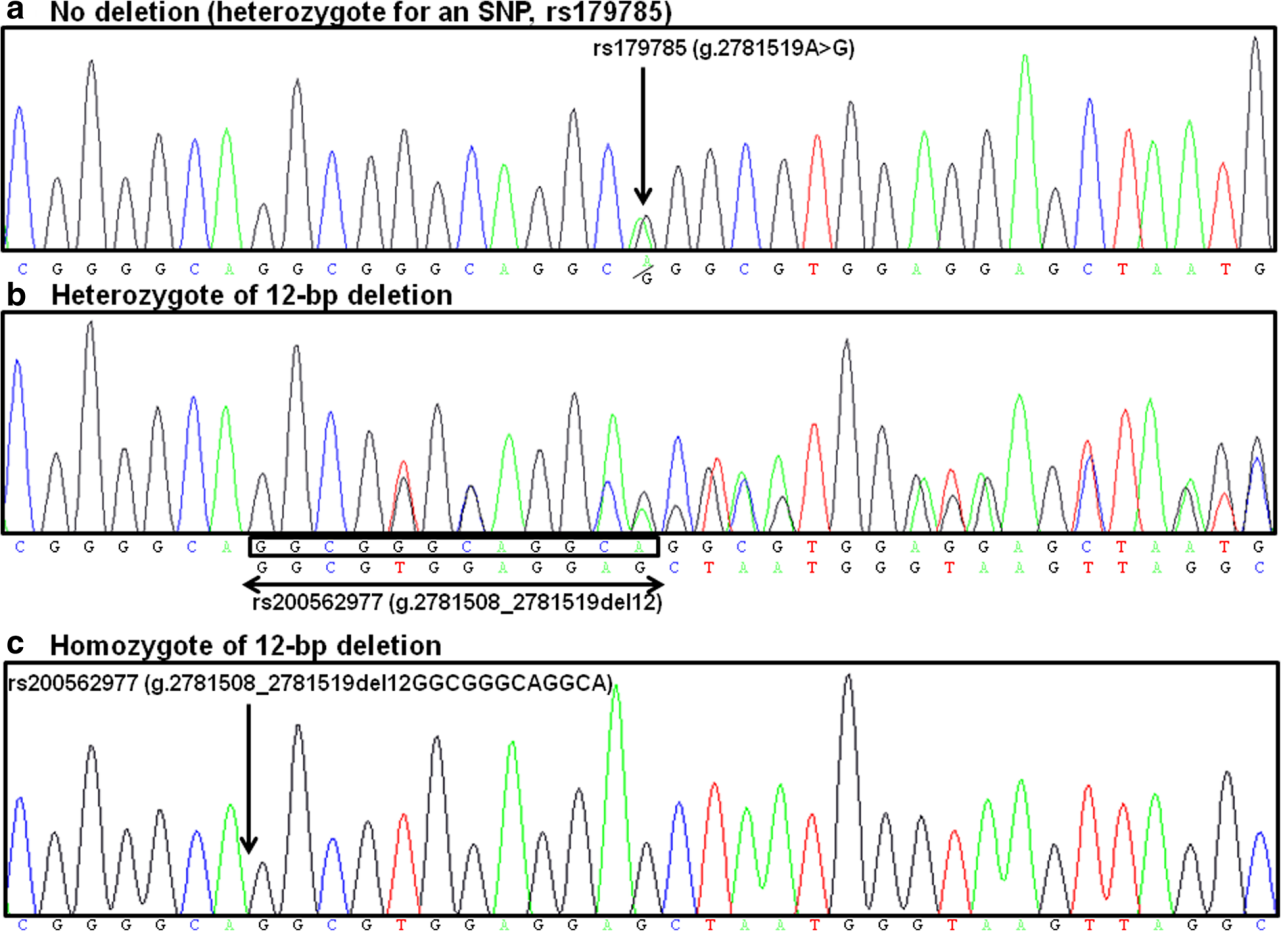
Common variants of *KCNQ1*, rs179785 and rs200562977, demonstrated by direct sequencing. rs200562977, a 12-bp deletion variant of *KCNQ1*, was identified as a common variant. rs179785 (A/G) is located at the last nucleotide (“A”) of 12-bp deletion site for rs200562977. Thus, when there is no deletion (**a**), rs179785 can be properly genotyped. On the other hand, when there is heterozygote (**b**) or homozygote (**c**) of 12-bp deletion, the position of rs179785 disappears

**Fig. 3:**
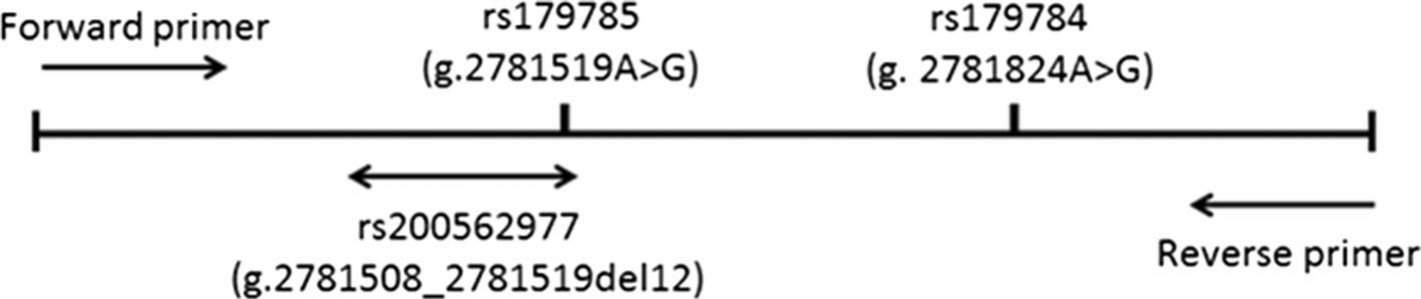
Location of three variants: rs179785, rs200562977 and rs179784 of *KCNQ1*. rs179785 (A/G) is located at the last nucleotide, “A”, of the 12-bp deletion site on rs200562977 (g.2781508_2781519del12GGCGGGCAGGCA). rs179784 is located 305 bp downstream from rs179785 and shows strong linkage disequilibrium with rs179785 (*D’* = 1.0 and *r*^*2*^ = 0.99). For direct sequencing of the three variants of *KCNQ1*, the primers were designed as follows: 5*’*-ACTTCCTGCCTCTGCTTTC-3*′* (forward primer) and 5*’*-TGAAGGAAGTGACCCCTG-3*′* (reverse primer), respectively

A representative plots of genotyping results by TaqMan method is shown in Fig. 1. Although allelic discrimination plots of rs12236871 of *RFX3* (Fig. 1a) and rs11653176 of *BCAS3* (Fig. 1b) are clearly divided into three groups for each genotype (major allele homozygote, heterozygote and minor allele homozygote), the plots of rs179785 of *KCNQ1 *are clustered into four groups, labeled as Groups 1, 2, 3 and 4 in Fig. 1c. Thus, to confirm the genotypes of samples of Groups 1 and 2, direct sequencing was performed to analyze the DNA sequence around rs179785 of *KCNQ1.* Although the genotypes of almost all the samples in Group 2 shown in Fig. 1c were heterozygous (A/G) for rs179785 (Fig. 2a), the heterozygous 12-bp deletion variant of *KCNQ1*, rs200562977 (Fig. 2b), was frequently found in Group 1 samples in Fig. 1c. Actually, rs179785 (A/G) is located at the last nucleotide (“A”) of this 12-bp deletion variant (rs200562977), as shown in Fig. 3. Further direct sequencing analysis revealed that Groups 3 and 4 also include a heterozygous 12-bp deletion variant, and several samples in Groups 2 and 4 exhibit a homozygous 12-bp deletion variant (Fig. 2c). These findings suggest that it is difficult to correctly genotype rs179785 of *KCNQ1* using the TaqMan or DNA micro-array method. Therefore, in subsequent analyses, rs179785 and rs200562977 of *KCNQ1* were genotyped by direct sequencing, not by the TaqMan method, in all samples. Moreover, by direct sequencing with the same primers, we were able to evaluate the genotypes of rs179784 of *KCNQ1*, which is located downstream from rs179785 by 305 bp (Fig. 3), and which shows strong LD with rs179785 (*D’* = 1.0 and *r*^*2*^ = 0.99).

All samples were successfully genotyped for the three variants of *KCNQ1* (rs179785, rs200562977 and rs179784) by direct sequencing. The call rates for the two SNPs (rs12236871 and rs11653176) by TaqMan method were more than 97.0%. All the variants were in Hardy-Weinberg equilibrium (*P* > 0.05). Table 1 shows the genotyping results of the three loci (*RFX3*, *KCNQ1* and *BCAS3*) for 723 clinically-defined gout patients and 913 controls. The common variant of *BCAS3*, rs11653176, showed a significant association with gout (*P* = 1.66 × 10^− 3^; odds ratio [OR] = 0.80; 95% confidence interval [CI]: 0.70–0.92). The direction of effect was the same as observed in the previous gout GWAS [8]. rs179785 and rs179784 of *KCNQ1* had a nominally significant association (*P* = 0.043 and 0.044; OR = 0.85 and 0.86; 95% CI: 0.73–0.99 and 0.75–1.00, respectively), but did not pass the significance threshold at *P* value < 0.017 (= 0.05/3) for multiple hypothesis testing using the Bonferroni correction. On the other hand, rs200562977 of *KCNQ1* and rs12236871 of *RFX3* did not show any significant association with gout.

**Table 1:** Association analysis of three loci with 723 clinically-defined gout cases and 913 controls

Variant^a^	Gene	Chr.	Position^b^	A1/A2	Genotypes						Alleles frequency model			
					Cases			Controls			Frequency^c^		*P* value	OR (95% CI)
					A1/A1	A1/A2	A2/A2	A1/A1	A1/A2	A2/A2	Cases	Controls		
rs12236871	*RFX3*	9	3589117	A/G	166	354	172	259	412	239	0.504	0.489	0.390	1.06 (0.92–1.22)
rs179785^d^	*KCNQ1*	11	2781519	A/G	196	277	102	204	379	143	0.418	0.458	0.043	0.85 (0.73–0.99)
rs200562977	*KCNQ1*	11	2781508–2781519	GGCGGGCAGGCA/−	575	142	6	726	173	14	0.107	0.110	0.744	0.96 (0.77–1.20)
rs179784	*KCNQ1*	11	2781824	A/G	288	331	104	304	474	135	0.373	0.407	0.044	0.86 (0.75–1.00)
rs11653176	*BCAS3*	17	59447369	C/T	227	346	148	218	476	219	0.445	0.501	1.66 × 10^− 3^	0.80 (0.70–0.92)

*Chr* chromosome, *OR* odds ratio, *CI* confidence interval

^a^dbSNP rs number. The variants of *KCNQ1* were genotyped by direct sequencing because of the presence of common deletion variant (rs200562977), whereas the variants of *RFX3* and *BCAS3* were correctly genotyped by the TaqMan method. In the analysis of rs179785 of *KCNQ1*, 148 cases and 187 controls with a heterozygous or homozygous deletion variant of rs200562977 were excluded because rs179785 is located at the last nucleotide, “A”, of rs200562977 (g.2781508_2781519del12GGCGGGCAGGCA). rs179784 of *KCNQ1* shows strong linkage disequilibrium with rs179785 (*D’* = 1.0 and *r*^*2*^ = 0.99)

^b^The positions of variants are based on NCBI human genome reference sequence Build 37

^c^‘Frequency’ means the frequency of A2

## Discussion

In this study, we were able, for the first time, to replicate the association between rs11653176 of *BCAS3* and gout. rs2079742, another intronic SNP of *BCAS3*, was previously reported to have an association with SUA level at the genome-wide significance level; however, it was not replicated in the same report [5]. BCAS3 is a coactivator of estrogen receptor alpha (ER-α) and is overexpressed in breast cancer cells [15], in which it is associated with tumor grade and proliferation [16]. The influence of sex hormones on SUA level is well known [17]. Especially, estradiol is thought to affect SUA levels through mechanisms modulating renal urate reabsorption and secretion. Increased SUA levels in postmenopausal women could be caused by the loss of estradiol. In addition, SUA levels decrease in postmenopausal patients using postmenopausal hormone compared with patients not using it [18]. Our findings suggest that risk allele (C) of rs11653176 of *BCAS3*, may increase renal urate reabsorption which results in increase of SUA levels and gout risk. Thus, although additional genetic and/or functional analyses will be necessary, the common variant of *BCAS3* might affect gout susceptibility in ways that are attributable to individual differences in responses to the effects of estrogen. Very recently, we have reported further GWAS of clinically-ascertained gout and identified 10 gout loci including *HIST1H2BF-HIST1H4E*, solute carrier family 17 member 1 (*SLC17A1*), solute carrier family 22 member 12 (*SLC22A12*), NIPA like domain containing 1 (*NIPAL1*) and family with sequence similarity 35 member A (*FAM35A*) [19]. Together with these gout loci, *BCAS3*, which is originally identified by the Chinese gout GWAS, will be very important for personalized genome medicine and/or prevention of gout.

## Conclusions

In summary, our present replication study demonstrated, as did a previous gout GWAS [8], an association between gout and the common variant of *BCAS3*. These findings suggest that the *BCAS3* locus is likely to have a common pathophysiological risk for gout.

## Abbreviations

ABCG2/BCRP: ATP-binding cassette transporter subfamily G member 2/breast cancer resistance protein
BCAS3: Breast carcinoma amplified sequence 3
ER-α: Estrogen receptor alpha
GLUT9/SLC2A9: Glucose transporter 9/solute carrier family 2 member 9
GWAS: Genome-wide association study
J-MICC Study: Japan Multi-Institutional Collaborative Cohort Study
JPT: Japanese in Tokyo
KCNQ1: Potassium voltage-gated channel subfamily Q member 1
LD: Linkage disequilibrium
OR: Odds ratio
RFX3: Regulatory factor X3
SNP: Single nucleotide polymorphisms
SUA: Serum uric acid

## References

[1] Matsuo H, Takada T, Ichida K, Nakayama A, Ikebuchi Y, Ito K (2009). Common defects of ABCG2, a high-capacity urate exporter, cause gout: a function-based genetic analysis in a Japanese population. Sci Transl Med.

[2] Woodward OM, Köttgen A, Coresh J, Boerwinkle E, Guggino WB, Köttgen M (2009). Identification of a urate transporter, ABCG2, with a common functional polymorphism causing gout. Proc Natl Acad Sci U S A.

[3] Matsuo H, Ichida K, Takada T, Nakayama A, Nakashima H, Nakamura T (2014). Common dysfunctional variants in ABCG2 are a major cause of early-onset gout. Sci Rep.

[4] Sulem P, Gudbjartsson DF, Walters GB, Helgadottir HT, Helgason A, Gudjonsson SA (2011). Identification of low-frequency variants associated with gout and serum uric acid levels. Nat Genet.

[5] Köttgen A, Albrecht E, Teumer A, Vitart V, Krumsiek J, Hundertmark C (2013). Genome-wide association analyses identify 18 new loci associated with serum urate concentrations. Nat Genet.

[6] Matsuo H, Yamamoto K, Nakaoka H, Nakayama A, Sakiyama M, Chiba T (2016). Genome-wide association study of clinically defined gout identifies multiple risk loci and its association with clinical subtypes. Ann Rheum Dis.

[7] Sakiyama M, Matsuo H, Nakaoka H, Yamamoto K, Nakayama A, Nakamura T (2016). Identification of rs671, a common variant of ALDH2, as a gout susceptibility locus. Sci Rep.

[8] Li C, Li Z, Liu S, Wang C, Han L, Cui L (2015). Genome-wide association analysis identifies three new risk loci for gout arthritis in Han Chinese. Nat Commun.

[9] Wallace SL, Robinson H, Masi AT, Decker JL, McCarty DJ, Yu TF (1977). Preliminary criteria for the classification of the acute arthritis of primary gout. Arthritis Rheum.

[10] The guideline revising committee of Japanese Society of Gout and Nucleic Acid Metabolism in Guideline for the Management of Hyperuricemia and Gout. 2nd ed. Guideline for the Management of Hyperuricemia and Gout. Osaka: Medical Review; 2010.

[11] Hamajima N, J-MICC Study Group (2007). The Japan multi-institutional collaborative cohort study (J-MICC study) to detect gene-environment interactions for cancer. Asian Pac J Cancer Prev.

[12] Sakiyama M, Matsuo H, Shimizu S, Chiba T, Nakayama A, Takada Y (2014). Common variant of leucine-rich repeat-containing 16A (LRRC16A) gene is associated with gout susceptibility. Hum Cell.

[13] R Development Core Team (2014). R. Foundation for Statistical Computing, Vienna.

[14] Abecasis GR, Altshuler D, Auton A, Brooks LD, Durbin RM, 1000 Genomes Project Consortium (2010). A map of human genome variation from population-scale sequencing. Nature.

[15] Barlund M, Monni O, Weaver JD, Kauraniemi P, Sauter G, Heiskanen M (2002). Cloning of BCAS3 (17q23) and BCAS4 (20q13) genes that undergo amplification, overexpression, and fusion in breast cancer. Genes Chromosomes Cancer.

[16] Gururaj AE, Singh RR, Rayala SK, Holm C, den Hollander P, Zhang H (2006). MTA1, a transcriptional activator of breast cancer amplified sequence 3. Proc Natl Acad Sci U S A.

[17] Adamopoulos D, Vlassopoulos C, Seitanides B, Contoyiannis P, Vassilopoulos P (1977). The relationship of sex steroids to uric acid levels in plasma and urine. Acta Endocrinol.

[18] Hak AE, Choi HK (2008). Menopause, postmenopausal hormone use and serum uric acid levels in US women--the third National Health and Nutrition Examination Survey. Arthritis Res Ther.

[19] Nakayama A, Nakaoka H, Yamamoto K, Sakiyama M, Shaukat A, Toyoda Y (2017). GWAS of clinically defined gout and subtypes identifies multiple susceptibility loci that include urate transporter genes. Ann Rheum Dis.

